# Assessment of a panel of tumor markers for the differential diagnosis of benign and malignant effusions by well-based reverse phase protein array

**DOI:** 10.1186/s13000-015-0290-4

**Published:** 2015-05-29

**Authors:** Till Braunschweig, Joon-Yong Chung, Chel Hun Choi, Hanbyoul Cho, Qing-Rong Chen, Ran Xie, Candice Perry, Javed Khan, Stephen M Hewitt

**Affiliations:** Laboratory of Pathology, National Cancer Institute, National Institutes of Health, Bethesda, MD 20892 USA; Institute of Pathology, RWTH Aachen University, Aachen, 52074 Germany; Department of Obstetrics and Gynecology, Samsung Medical Center, Sungkyunkwan University School of Medicine, Seoul, 135-710 Republic of Korea; Department of Obstetrics and Gynecology, Gangnam Severance Hospital, Yonsei University College of Medicine, Seoul, 135-720 Republic of Korea; Oncogenomics Section, Pediatric Oncology Branch, National Cancer Insititute, National Institutes of Health, Bethesda, MD 20892 USA; Present affiliation: Center for Biomedical Informatics and Information Technology, National Cancer Institute, National Institutes of Health, Bethesda, MD 20892 USA; Antibody Characterization Laboratory, Advanced Technology Program, Leidos Biomedical Research, Inc., Frederick, MD USA

**Keywords:** Cytodiagnosis, Tumor biomarkers, Neoplasms, Mucin 1, Epithelial membrane antigen, Pleural effusion

## Abstract

**Background:**

The differential diagnosis of benign and malignant effusion is often hampered by low cell content or insufficiently preserved tumor cells. In this study, we evaluated the combined diagnostic value of six tumor markers measured by well-based reverse-phase protein array (RPPA) for diagnosis of malignant effusion.

**Methods:**

A total of 114 patients (46 with malignant effusions, 32 with probable malignant effusions, and 36 with benign effusions) were enrolled. Expressional levels of MUC1, EMA, Pan-CK, HSP90, TGF-β and CA125 were determined by well-based RPPA.

**Results:**

Median relative expression of MUC1, Pan-CK and EMA were significantly higher in malignant effusion than those in probable malignant or benign (*p* < 0.001, *p* = 0.003, *p* < 0.001, respectively), whereas the level of TGF-β in malignant effusions were significantly lower than that in the other groups (*p* = 0.005). For predicting malignancy, EMA presented the best areas under the curve of 0.728 followed by MUC1 of 0.701. The sensitivity of 52.0% for MUC1 and 48.0% for EMA were not better than cytology. However, sensitivity, negative predictive value, and accuracy of the tumor marker panel were better than cytology by 14.7%, 7.5%, and 6.1%, respectively.

**Conclusions:**

Tumor marker panel measured by well-based RPPA showed values in the differential diagnosis between benign and malignant effusions. Further large scale studies need to be performed to evaluate the utility of this panel of markers.

**Virtual slides:**

The virtual slide(s) for this article can be found here: http://www.diagnosticpathology.diagnomx.eu/vs/1433424467160224

**Electronic supplementary material:**

The online version of this article (doi:10.1186/s13000-015-0290-4) contains supplementary material, which is available to authorized users.

## Background

Effusions are common complications in patients with advanced or metastatic tumors, but also seen in other diseases, e.g. liver or heart failure or kidney malfunction. It is important to differentiate between benign and malignant effusions for rational decision making in choosing the therapeutic strategy and assessing patients’ prognosis. Although cytopathological examination is considered a standard method for the diagnosis of effusions, several challenging issues remain in detecting malignant cells in the effusion, including low sensitivity [1–3]. Furthermore, the sensitivity is dependent on the abundance of morphologically intact cells and experience of the cytopathologist. Especially in pleural effusion, the difficulty lies in separating normal or reactive mesothelial cells from cells of malignant mesothelioma or carcinoma. Another limitation is that current standard cytological examination is often unable to distinguish between different types of malignant cells without the use of special additional studies such as immunocytochemistry (ICC) or immunohistochemistry (IHC) on cell blocks [4]. ICC improves the diagnostic accuracy of conventional cytology [5], but there is no agreement on the ideal combination of immune markers used. In this context, protein content or tumor markers within the fluid have been targeted as additional diagnostic discriminators, including β-HCG [6], albumin [7, 8], vascular permeability factor (VPF) [9], transthyretin [10], CEA [11], CA19-9 [12], EZH2 [13] and calretinin [14]. In addition to these single protein approaches, panels combining different markers have also been proposed [15, 16]. These studies show a high interest in being independent of variable cell content in effusions. Unfortunately, none of the makers or panels has shown sufficient sensitivity and specificity to be considered as a potential diagnostic marker of effusions.

Mucins are proteins of high molecular weight above 100 kDa, and, up to now, 20 different mucins with some isoforms have been identified. Among them, mucin 1 (MUC1) seems to be the most studied and promising tumor marker and recently, it was described as a target for therapy [17]. Epithelial membrane antigen (EMA) has been applied as an immunocytological marker in distinction of carcinoma cells versus mesothelioma cells [18, 19], while the descriptive name implies its primary lack of detailed characterization of amino acid sequence or primary coding gene. Confirming this fact, later studies in 1996 showed that the monoclonal antibody to EMA binds at the same marker protein as CA15-3 and MUC1 [20].

Reverse phase protein array (RPPA) is a sensitive and high throughput technology that uses a sandwich format for antigen capture. Over the last decade, RPPA has been applied to a diverse range of sample types including serum/plasma samples [21, 22]. Here, we used this assay to assess the expression of MUC1 and other markers (EMA, Pan-CK, HSP90, TGF-β and CA125) used in immunocytochemistry in ascites and pleural fluid. The aim of this study was to determine the diagnostic values of combination markers, and with this well-based RPPA methodology, we expected to demonstrate better distinction of malignant from benign effusions, independent of primary tumors and independent of cell content.

## Methods

### Sample collection

The study subjects consisted of 114 effusion samples, 38 ascites and 76 pleura effusions. Fresh effusion specimens were collected and centrifuged at × 3000 g for 10 min at 4 °C. The supernatant was stored at −80 °C until the well-based RPPA was performed. Study samples were classified into three groups according to the etiology of the effusion: malignant, probable malignant and benign effusion. An effusion was categorized as malignant if malignant cells were found in effusion fluid or in the biopsy specimen tissue. A diagnosis of probable malignant effusion was made if the effusion fluid cytology was negative in patients with a known history of a primary malignancy, after ruling out benign causes of the effusion. The effusion was classified as benign when no malignant cells were found and no history of a malignant tumor was known [23]. All effusion samples were collected as pseudonymized samples for proteomic studies, which was approved by the institutional review board of the RWTH Aachen University Hospital (approval no. ek 173/06). This study was additionally approved by the Office of Human Subjects Research at the National Institutes of Health.

### Well-based reverse phase protein array

Protein concentrations of effusion samples were measured by standard procedures, using the BCA Protein Assay kit (Pierce Biotechnology, Rockford, IL). Expressional signals of MUC1, EMA, Pan-CK, HSP90, TGF-β, CA125, and glyceraldehyde-3-phospahte dehydrogenase (GAPDH) were determined by well-based RPPA, by means of electrochemiluminescence immunoassay [24–26]. The signal of each marker was normalized for GAPDH signal as in western blotting. Briefly, five microliters (400 ng/μl) of native sample were added to Meso Scale Discovery (MSD, Gaithersburg, MD) Multi-Spot™ plates (MA2400 96 HB Plate). The plate was dried at room temperature for 90 min, and the plate was subsequently incubated at 37 °C for 30 min. The antigen-coated plates were blocked with 5% nonfat dry milk in PBST for 60 min at room temperature and further incubated with anti-MUC1 (diluted 1:1000; Mouse monoclonal, clone# MA695, Novocastra, Buffalo Grove, IL), anti-EMA (diluted 1:1000; Mouse monoclonal, clone# E29, Dako), anti-Pan-CK (diluted 1:500; Rabbit polyclonal, Dako), anti-HSP90 (diluted 1:500; Rabbit polyclonal, Cell Signaling), anti-TGF-β (diluted 1:500; Rabbit polyclonal, Cell Signaling, Denvers, MA), anti-CA125 (diluted 1:500; Dako, Carpinteria, CA) or anti-GAPDH (diluted 1:1000; Mouse monoclonal, Calbiochem, Gibbstown, NJ) in PBST containing 5% BSA at 4 °C overnight, followed by 3 washes with PBST. The plates were incubated for 60 min with goat anti-mouse or anti-rabbit SULFO-TAG™ antibodies at a dilution of 1:2000 (0.5 μg/ml) with 5% nonfat dry milk in PBST. The plates were then aspirated and washed three times with PBST. Finally, MSD-T read buffer was added to the plates and they were read on the MSD Sector Imager 2400 reader (Meso Scale Discovery). BSA coated wells were included on each plate as a negative control for non-specific binding effects. The values from non-specific wells were subtracted from all standard samples to calculate actual value. Two independent experiments were performed with triplicates.

### Western blotting

Equal protein amounts (50 μg) of each sample were resolved by 4-12% NuPAGE® Novex Bis-Tris polyacrylamide gel, and electroblotted to nitrocellulose membrane using iBlot™ Dry Blotting System (Invitrogen, Carlsbad, CA). The membranes were blocked with 5% nonfat dry milk in TBST for 60 min, washed, and subsequently incubated overnight at 4 °C in TBST (50 mM Tris, pH 7.5, 150 mM NaCl, 0.05% Tween-20) with 5% BSA containing the following antibodies; anti-MUC 1 (diluted 1:1000; Mouse monoclonal, Novocastra), anti-EMA (diluted 1:1000; Mouse monoclonal, DAKO) or anti-GAPDH (diluted 1:5000; Mouse monoclonal, Calbiochem). Specific bindings were detected with horseradish peroxidase-labeled anti-mouse antibodies (Chemicon International, Temecula, CA) and enhanced with SuperSignal Chemiluminescence kit (Pierce Biotechnology). Signals were detected on KODAK BIOMAX MR X-ray film (Kodak, Rochester, NY).

### Statistical analysis

The boxplot was used to present the distribution of tumor markers in effusions. For comparisons between groups, Chi-square test for categorical variables and the nonparametric Kruskal-Wallis or Mann–Whitney U test for continuous variables were used. Receiver operating characteristic analysis was used to compare the diagnostic accuracy of different tumor markers. Cut-off values were selected to maximize the specificity in the diagnosis of malignant effusion. The combination of tumor markers was used in a parallel manner with an “or” rule, wherein the test result was considered positive if the cut-off point for any of the markers was exceeded. For all analyses, a *P* value < 0.05 was considered significant. All analyses were performed using SPSS version 21.0 (SPSS Inc., Chicago, IL).

The hierarchical clustering analysis was performed using R to visualize the tumor marker expression pattern and to cluster the effusion samples based on the score of seven antibodies. The pearson uncentered correlation was used as distance metric with average linkage. Three main clusters of samples were evaluated for an association with clinicopathological factors using chi-square test.

## Results

### Etiology and protein concentration of effusion

The specific etiologies of effusions are presented in Additional file 1: Table S1. Of the 114 cases, 46 (40.4%) samples (15 ascites and 31 pleural effusions) were malignant effusions, 32 (28.1%) samples (12 ascites and 20 pleural effusions) were probable malignant effusions, and 36 (31.6%) samples (11 ascites and 25 pleural effusions) were benign effusions. In malignant ascites, ovarian carcinoma was the most frequent primary (10/15, 66.6%), and in malignant pleura effusions, non-small cell lung carcinoma was predominant (19/31, 61.3%). The benign samples were from patients with heart failure (10/36, 27.8%), liver cirrhosis (16/36, 44.4%), and inflammatory diseases (10/36, 27.8%).

**Fig. 1:**
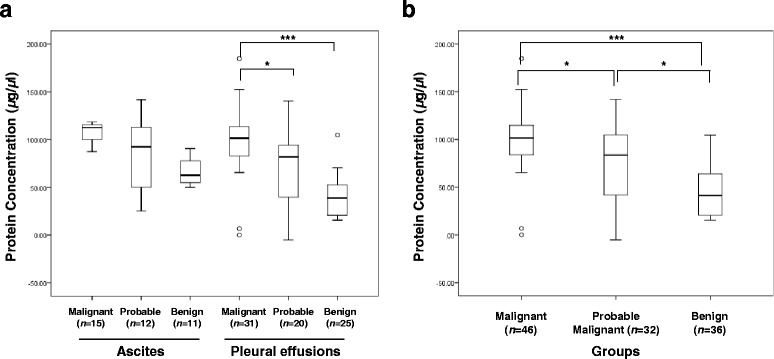
Box plots of protein concentration among benign, probable malignant and malignant cases. The protein concentrations are displayed by samples origin (**a**) and disease category (**b**). The protein concentrations of cytological positive specimens are higher than negative cases. Furthermore, the protein concentration of malignant case is higher than benign (*p* < 0.001) or probable malignant cases (*p* = 0.032). *, *p* < 0.05; **, *p* < 0.01; ***, *p* < 0.001

As expected, the protein concentrations were 1.63-fold higher in effusion specimens positive for malignancy by cytological examination compared to those negative by cytology (mean 81.0 vs 49.8, respectively, *p* < 0.001). This pattern was observed in both ascites and pleural effusions (*p* < 0.023) but ascites did not reach statistical significance due to small size of specimen number (Fig. 1a). By diagnosis criteria, the protein concentration in malignant effusion and probable malignant effusion were higher than that in benign effusion (mean 98.3 vs 46.4, *p* < 0.001 and 77.7 vs. 46.4, *p* = 0.015, respectively) (Fig. 1b).

### Tumor marker assessment by well-based RPPA

**Fig. 2:**
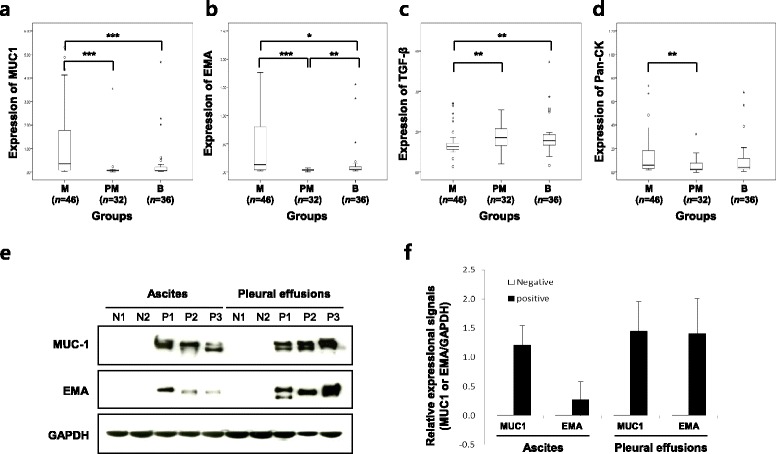
Assessments of MUC1, EMA, TGF-β, and Pan-CK expressions by well-based reverse phase protein array and western blotting. Relative expressions of MUC1 **(a)**, EMA **(b)**, TGF-β **(c)** and Pan-CK **(d)** were categorized by samples. Relative expressional signals are displayed as a ratio of each protein to GAPDH. MUC1 and EMA expressions of malignant cases are significantly higher than benign cases (*p* = 0.001 and *p* = 0.011, respectively). **(e)** MUC1 and EMA protein levels were analysed by western blot. GAPDH is included as an internal loading control. **(f)** The bar graph shows the average ± SD of two independent experiments. M, malignant; PM, probable malignant; B, benign; N, negative cytology; P, positive cytology; *, *p* < 0.05; **, *p* < 0.01; ***, *p* < 0.001

To assess the level of the six tumor markers (MUC1, EMA, Pan-CK, HSP90, TGF-β and CA125), we performed the well-based RPPA with all ascites and pleural effusions. Results of tumor markers quantitation by the well-based RPPA are summarized in Table 1. Median relative expressions of MUC1, Pan-CK and EMA were significantly higher in malignant effusion than those in probable malignant or benign (*p* < 0.001, *p* = 0.003, *p* < 0.001, respectively), whereas the level of TGF-β were significantly lower in malignant effusions than that in the other groups (*p* = 0.005) (Table 1). Notably, expressional signals of MUC1 and EMA in malignant were significantly higher than benign (*p* = 0.001 and *p* = 0.011, respectively) (Fig. 2a and b). The elevated expressions of MUC1 and EMA were confirmed by western blotting (Fig. 2e and f). The high expressions of both proteins were prominent in cells from both ascites and pleural effusions. HSP90 and CA125 failed to demonstrate differences based on diagnosis.

**Table 1:** Median titers of tumor markers in study groups

Tumor Markers^a^	Malignant (*n* = 46)	Probable Malignant^b^ (*n* = 32)	Benign (*n* = 36)	*P* value^c^
**MUC1**	0.355 (0.081-1.830)	0.073 (0.043-0.096)	0.074 (0.046-0.217)	< 0.001
**EMA**	0.131 (0.043-0.810)	0.038 (0.030-0.050)	0.052 (0.038-0.095)	< 0.001
**Pan-CK**	0.059 (0.031-0.188)	0.024 (0.017-0.080)	0.039 (0.025-0.118)	0.003
**HSP90**	0.206 (0.167-0.258)	0.178 (0.133-0.231)	0.184 (0.148-0.246)	0.172
**TGF-β**	0.128 (0.113-0.145)	0.171 (0.132-0.220)	0.156 (0.133-0.186)	0.005
**CA125**	0.149 (0.064-0.269)	0.121 (0.057-0.213)	0.148 (0.070-0.242)	0.258

Data are presented as median (quartiles)

^a^After normalization with GAPDH level, relative expressional signals were represented as a ratio (tumor marker/GAPDH)

^b^No malignant effusion but history of malignancy

^c^Significance level of Kruskal-Wallis test

### Hierarchical clustering and correlations between biomarkers

**Fig. 3:**
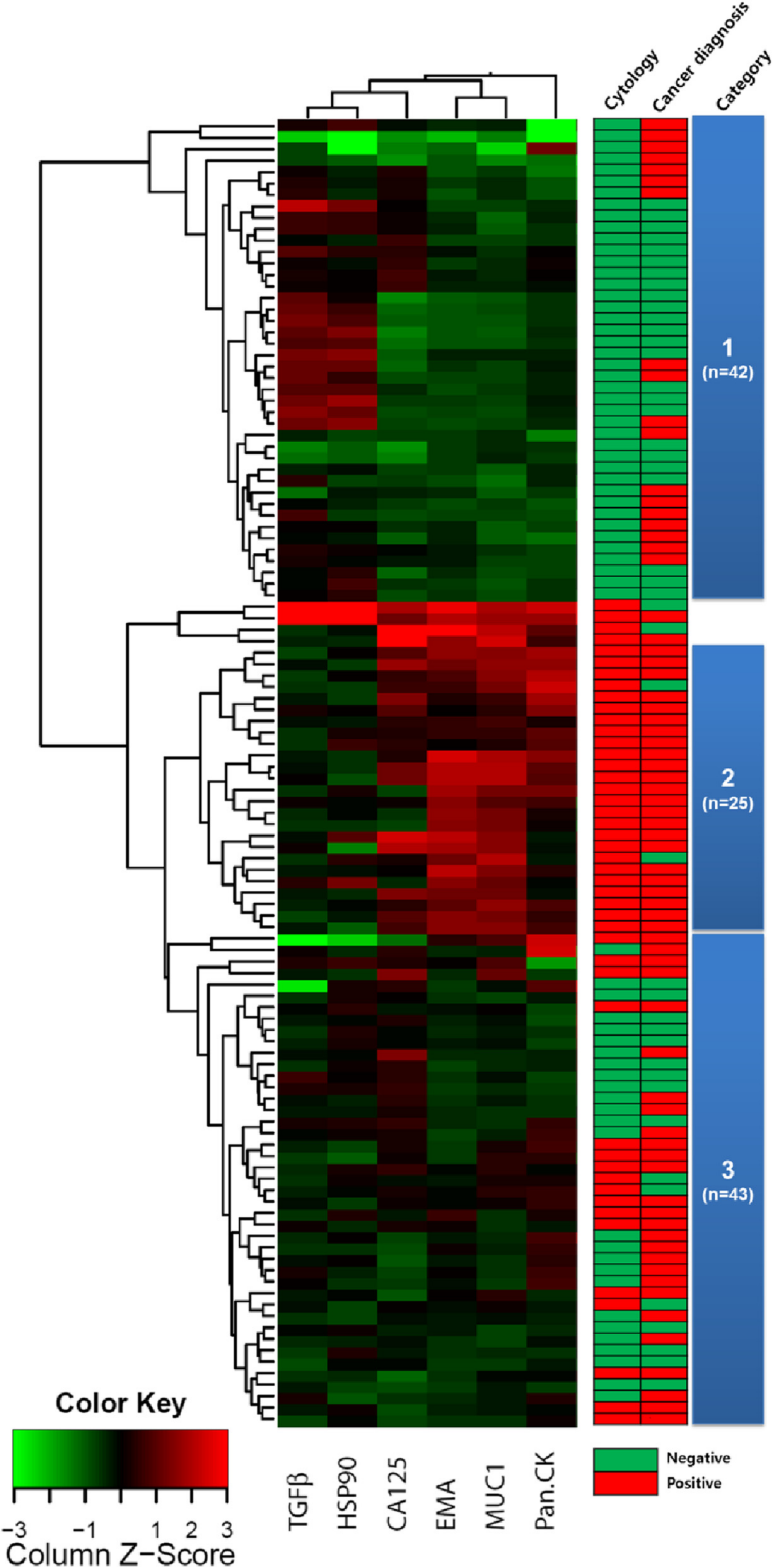
Hierarchical clustering and multidimensional scaling analysis. Each row represents a sample, and each column represents a protein. A pseudo-colored representation of the ratio (log2-transformed and z-scored across the samples) is shown. On the bottom are the symbols of 6 proteins. On the right are the effusion fluid cytology (negative or positive), cancer diagnosis (negative or positive), and the category of three main clusters

To find the clustering of samples according to markers, a total of 114 cases were analysed by hierarchical clustering with the level of each marker. As shown in Fig. 3, three main groups could be categorized with four samples not included in any category. Category 1 consists exclusively of negative cytology and category 2 exclusively of positive cytology. Category 3 represents intermediate of category 1 and 2. The majority of cases of category 2 have higher expression of MUC1, EMA, Pan-CK, and CA125 that in the other group. In contrast, category 1 represent the lowest average activity in three (MUC1, EMA, and Pan-CK) of the six markers assayed. In addition, the etiology of effusion was different according to categories classified (Table 2). Colon cancer was classified predominantly as category 1 (60.0%), whereas ovarian cancer was categorized predominantly as category 2 (64.3%). Of the benign etiology, liver cirrhosis was classified predominantly to category 1 (81.3%), while heart insufficiency and inflammatory disease were equally categorized to 1 and 3, respectively.

**Table 2:** Association between clinicopathological characteristics and three groups defined with cluster analysis

		**Category** ^**a**^		***P*** **value**
	**1 No. (%)**	**2 No. (%)**	**3 No. (%)**	
**Group**	42	25	43	
Malignant	0 (0.0)	25 (59.5)	17 (40.5)	<0.001
Probable malignant^b^	19 (59.4)	0 (0.0)	13 (40.6)	
Benign	23 (63.9)	0 (0.0)	13 (36.1)	
**Fluid**				
Ascites	19 (50.0)	8 (21.1)	11 (28.9)	0.155
Pleural effusion	23 (31.9)	17 (23.6)	32 (44.4)	
**Cytology result**				
Positive	0 (0.0)	25 (59.5)	17 (40.5)	<0.001
Negative	42 (61.8)	0 (0.0)	26 (38.2)	
**Etiology (cancer)**				
Breast	2 (33.3)	2 (33.3)	2 (33.3)	<0.001
Colon	9 (60.0)	1 (6.7)	5 (33.3)	
Lung	4 (15.4)	11 (42.3)	11 (42.3)	
Ovary	0 (0.0)	9 (60.0)	6 (40.0)	
Others	4 (30.8)	2 (15.4)	7 (53.8)	
**Etiology**				
Heart failure	5 (55.6)	0 (0.0)	4 (44.4)	0.002
Liver cirrhosis	13 (81.3)	0 (0.0)	3 (18.8)	
Inflammatory	5 (50.0)	0 (0.0)	5 (50.0)	

^a^The remaining 4 cases were not categorized into any grouping

^b^No malignant effusion but history of malignancy

### Operating characteristics of tumor markers predicting malignancy

**Fig. 4:**
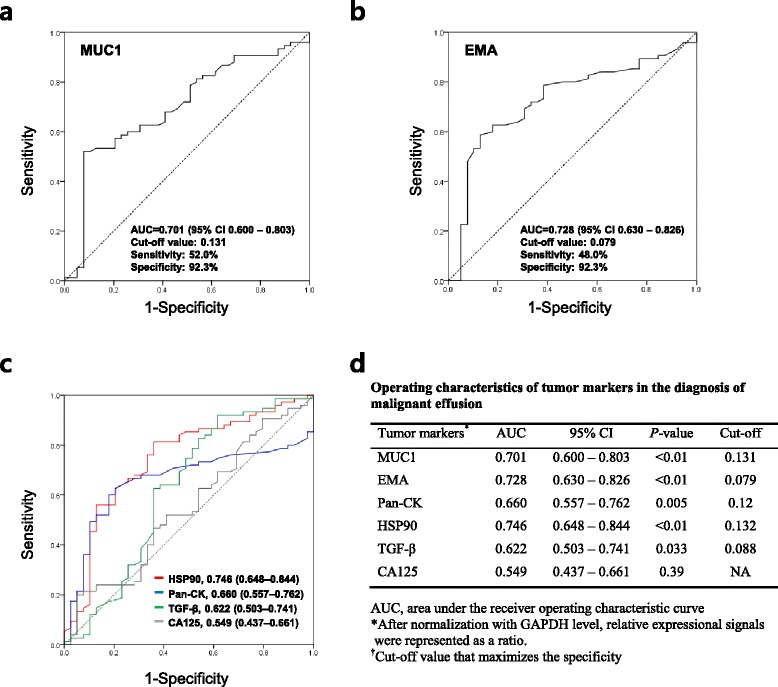
Receiver operating characteristic (ROC) curves. The ROC curves were plotted depending on MUC1 **(a)**, EMA **(b)**, and other tumor markers (HSP90, Pan-CK, TGF-β, and CA125) **(c).** Operating characteristics of six tumor markers for malignant effusion diagnosis **(d)**. Relative expressional signals were represented as a ratio after normalization with GAPDH

Lastly, receiver operating characteristic (ROC) curves were plotted to see the diagnostic accuracy of each marker for the diagnosis of malignancy (Fig. 4). The areas under curve (AUC) did not reveal a single marker as significantly superior. Of the markers, HSP90 showed the best AUC of 0.746 (95% CI, 0.503 – 0.741) and followed by EMA of 0.728 (95% CI, 0.630 – 0.826) and MUC1 of 0.701(95% CI, 0.600 – 0.803). Interestingly, CA125 showed no diagnostic value in this methodology. The cut-off values of the five tumor markers (MUC1, EMA, Pan-CK, HSP90 and TGF- β) were determined that best differentiated benign from malignant effusions with the utmost specificity (Fig. 4d). The sensitivity, specificity, positive predictive value (PPV) and negative predictive value (NPV), and accuracy for selected cut-off points for each tumor marker are shown in Table 3. The sensitivity and specificity of cytology for the diagnosis of malignancy was 57.3% and 92.3%, respectively. Notably, the diagnostic value of combined MUC1 and EMA was the same with cytology examination (Table 3). Furthermore, the sensitivity, NPV, and accuracy of the combined panel of five markers were better than cytology by 14.7%, 7.5%, and 6.1%, respectively.

**Table 3:** Diagnostic accuracy of each analysed markers

Tumor markers	Sensitivity (%)	Specificity (%)	PPV (%)	NPV (%)	Accuracy (%)
**Cytology**	57.3	92.3	93.5	52.9	69.3
**MUC1**	52.0	92.3	92.9	50.0	65.8
**EMA**	48.0	92.3	92.3	48.0	63.2
**Pan-CK**	36.0	92.3	90.0	42.9	55.3
**HSP90**	14.7	92.3	78.6	36.0	41.2
**TGF-β**	4.0	92.3	50.0	33.3	34.2
**MUC 1 + EMA**	57.3	92.3	93.5	52.9	69.3
**MUC 1 + EMA+ Pan-CK**	64.0	89.7	92.3	56.5	72.8
**MUC 1 + EMA+ Pan-CK + HSP90**	70.7	82.1	88.3	59.3	74.6
**MUC 1 + EMA+ Pan-CK + HSP90 + TGF-β**	72.0	82.1	88.5	60.4	75.4

PPV, positive predictive value; NPV, negative predictive value

## Discussion

Effusions in pleura or abdomen can hamper body functions in a very severe way resulting in e.g. respiratory failure, heart failure or fluid balance. Diagnostic evaluation of effusions to determine and fight the cause, next to mechanical relief is of major interest in planning general or symptomatic therapy.

In order to evaluate the diagnostic values of putative effusion tumor markers within the protein content in the fluid compartment, we measured MUC1, EMA, Pan-CK, HSP90, TGF-β and CA125 by a well-based RPPA methodology in pleural effusions and ascites. This methodology is based on antibody binding and measures the binding by extremely sensitive electroluminescence to cover even very low protein concentrations. MUC1 and EMA levels were significantly higher in patients with malignant effusions than those in benign effusions. Analysis of the ROC curves showed that EMA presented the best AUC of 0.728 followed by MUC1 of 0.701 for the diagnosis of malignant effusions. Although single markers were less sensitive than cytopathology (52.0 for MUC1and 48.0 for EMA), combined markers showed higher sensitivity and accuracy. In addition, adding the panel of tumor markers to the cytological analysis improved the sensitivity, NPV, and accuracy by 14.7%, 7.5%, and 6.1%, respectively, compared to cytopathological examination alone. To the best of our knowledge, diagnostic utility of MUC1 and EMA measured by well-based RPPA have not been previously determined in pleural effusion or ascites.

Body fluid cytopathology has traditionally been the analytical method of choice for the detection of tumor cells, as reflected in the International Union Against Cancer/American Joint Committee on Cancer tumor-node metastasis system [27]. However, in approximately 40% of cases, the cytological analysis does not provide a decisive answer as to whether the effusion is of malignant cause or not [1–3]. In addition, the sensitivity is primarily dependent on the experience of the cytologist, the number of morphologically intact tumor cells, and the amount of material submitted [28–31]. One recent study has suggested that current clinical guidelines recommending a submission of 50 ml of pleural fluid may be suboptimal [30]. Immunocytochemistry (ICC) may complement cytology, but its utility in differentiating between benign pleural disease and mesothelioma is somewhat controversial [32]. Building “cell blocks” or “cyto blocks” out of effusions by embedding the cell content in agarose gel and build paraffin blocks for subsequently proceed with IHC requires a good cell content [4, 33]. Cytopathology itself is a quick analysis, broadening diagnostic approaches to immunocytochemistry or building cell blocks are time consuming. These limitations of cytological diagnosis of effusions drive the continual search for novel diagnostic auxiliaries. Such a test can potentially negate the need for otherwise ascertaining invasive procedures and provide rapid definitive diagnosis.

This RPPA is an antibody-based proteomic technology which is suitable for profiling of signaling protein’s expression and modification in low abundance [34, 35]. It allows concomitantly monitoring of the expression of particular protein in hundreds of samples in a quantitative manner. In addition, the advantages of high throughput, sensitivity and cost effectiveness of RPPA have accelerated the incorporation of this technology in basic, preclinical and clinical research areas [36]. Especially in the upcoming field of “liquid biopsy”, in which solved contents in body fluids (e.g. proteins, DNA) are in focus to be markers for diagnosis or prognosis [37]. Although remarkable advances in the RPPA platform have extended into the proteomic research area, there are still limitations to the full strength of this methodology, including sophisticated printer requirement and complicated study design. In this context, we have established a well-based RPPA platform with technical improvements. Basically, our well-based RPPA platform does not require the use of a printer arrayer, nor does it require scoring of a dilution curve [24, 25]. Furthermore, one substantial advantage is its capacity to measure multiple proteins and develop a normalized metric based on the expression of a largely invariant protein. This platform has been successfully implemented into the Antibody Portal of NCI (http://antibodies.cancer.gov) as an antibody validation tool for new mAbs [26]. As most proteomic assays, this methodology is also mainly dependent on the antibody quality.

A large number of studies on the potential diagnostic usefulness of effusion fluid tumor markers have been published, which report either encouraging [38, 39] or disappointing results [40–42]. These disagreements can be attributed to different factors, including the heterogeneity of tumor types, or the use of different methodologies and cut-off values in assays, among others. In this study, we used a well-based RPPA with GAPDH protein signal as an internal control to measure possible degradation of the protein content from each sample. This methodology shows an advantage that can evaluate tumor marker level without the risk of low reliability and poor validity [24, 25].

In recent years, there have been many tumor markers that were used in the differential diagnosis of effusions. However, no tumor marker alone had the sufficient diagnostic accuracy in discriminating malignant from benign effusions. In addition, the clinical value of their combined use has been evaluated with enhanced prediction of malignancy. On the other hand, the use of combinations is always followed by a decrease in specificity. In this study, the combination of five tumor markers had the maximum sensitivity and accuracy with acceptable specificity. Even without definite diagnosis, these combined tumor markers may at least aid in selecting patients when carrying out a more invasive procedures.

In OMIM (online mendelian inheritance for men) database for proteins by the National Center for Biotechnology Information, three synonyms for one protein, MUC1 are named: Peanut-Reactive Urinary Mucin, PUM; Tumor Associated Epithelial Polymorphic Epithelial Mucin, PEM; Epithelial Membrane Antigen, EMA. Two others can be found in literature: CA 15–3 and Episialin [20]. Basically, MUC1 and EMA antibodies target the same protein. EMA was first described in 1981 as an antiserum that was made using a cell extraction [43]. EMA was therefore not made with the knowledge of using a certain binding area of MUC1. In this study, we used murine monoclonal antibodies (E29), which were produced using milk-fat-globule-membranes as an antigen, established in 1985 and frequently applied in research [44]. Unfortunately, the specific binding site of EMA is still unknown. On the other hand, the MUC1 antibodies (MA695) are precisely described to recognize a sialylated carbohydrate antigen on MUC1 mucin [45]. It is likely that both antibodies bind to different epitopes. Due to different degradation statuses of MUC1 molecule or fragmented secretion in an effusion, different expression levels of MUC1 and EMA are expected. In addition, Langner et al. tested and compared MUC1 (MA695) and EMA (E29) antibodies using immunohistochemistry, not by western and reverse-phase protein array [46]. Antibody avidity mainly relies on the proteomic technology used. Thus, we chose to incorporate MUC1 and EMA by well-based RPPA to the existing tumor panel. Noting the various binding affinities of single target proteins to multiple epitopes of one protein, it is beneficial to use a panel of monoclonal antibodies of different clones covering different epitopes for more sensitive analysis.

## Conclusions

In conclusion, we demonstrated that effusion fluid tumor markers (MUC1, EMA, Pan-CK, HSP90 and TGF-β) measured by well-based RPPA have a limited albeit not a negligible value in the workup of effusions. The combined assay of five tumor markers is helpful in increasing the sensitivity and accuracy in diagnosing malignant effusions. Further large scale studies need to be performed to ensure whether this panel of tumor marker can replace or become an alternative to other markers in patients with metastatic disease.

## Additional files

Additional file 1: Table S1. Etiology of the effusions.

## Abbreviations

AUC: Areas under curve
EMA: Epithelial membrane antigen
GAPDH: Glyceraldehyde-3-phospahte dehydrogenase
ICC: Immunocytochemistry
IHC: Immunohistochemistry
MUC1: Mucin 1
NPV: Negative predictive value
OMIM: Online mendelian inheritance for men
PPV: Positive predictive value
ROC: Receiver operating characteristic
RPPA: Reverse-phase protein array
VPF: Vascular permeability factor
