# Supplementary material for: Assessment of a panel of tumor markers for the differential diagnosis of benign and malignant effusions by well-based reverse phase protein array
Source: Diagn Pathol. 2015 May 29;10:53. doi: 10.1186/s13000-015-0290-4 (PMC4447024; doi:10.1186/s13000-015-0290-4)
Supplement: Supplementary file 1 — Etiology of the effusions. [file 13000_2015_290_MOESM1_ESM.pdf]

**Supplemental Table 1 Etiology of the effusions (*n*=114)**

| Etiology | Number of cases | | |
|---|---|---|---|
| | **Ascites No. (%)** | **Pleural effusions No. (%)** | **Total No. (%)** |
| **Malignant** | 15 (13.2) | 31 (27.2) | 46 (40.4) |
| Breast | 0 | 3 | 3 |
| Bronchus | 0 | 3 | 3 |
| Uterine cervix | 1 | 0 | 1 |
| Colon | 1 | 1 | 2 |
| Mesothelioma | 0 | 1 | 1 |
| NHL | 1 | 0 | 1 |
| NSCLC | 0 | 13 | 13 |
| Ovary | 10 | 4 | 14 |
| Prostate | 0 | 2 | 2 |
| Small cell lung cancer | 0 | 3 | 3 |
| Uterine corpus | 0 | 1 | 1 |
| Unclear primary | 2 | 0 | 2 |
| **Probable malignant** [a] | 12 (31.6 ) | 20 (17.5) | 32 (28.1) |
| Breast | 0 | 3 | 3 |
| Bronchus | 0 | 2 | 2 |
| Colon | 8 | 4 | 12 |
| Esophagus | 0 | 1 | 1 |
| Mesothelioma | 0 | 3 | 3 |
| NSCLC | 0 | 6 | 6 |
| Ovary | 1 | 0 | 1 |
| Pancreas | 1 | 0 | 1 |
| Rectus | 0 | 1 | 1 |
| Uterine corpus | 2 | 0 | 2 |
| **Benign** | 11 (28.9 ) | 25 (21.9) | 36 (31.6) |
| Heart insufficiency | 0 | 10 | 10 |
| Liver cirrhosis | 11 | 5 | 16 |
| Pneumonia | 0 | 7 | 7 |
| Septic fever | 0 | 3 | 3 |
| **Total** | 38 (33.4) | 76 (66.6) | 114 (100) |

[a] No malignant effusion but history of malignancy
